# Supplementary material for: A Large-Scale High-Throughput Screen for Modulators of SERCA Activity
Source: Biomolecules. 2022 Nov 30;12(12):1789. doi: 10.3390/biom12121789 (PMC9776381; doi:10.3390/biom12121789)

A Large-Scale High-Throughput Screen for Modulators of SERCA Activity

Philip A. Bidwell [1], Samantha L. Yuen [2], Ji Li [2], Kaja Berg [3], Robyn T. Rebbeck [2], Courtney C. Aldrich [3], Osha Roopnarine [2], Razvan L. Cornea [2] and David D. Thomas [2,*]

[1] Department of Medicine, Cardiovascular Division, University of Minnesota, Minneapolis, MN 55455, USA
[2] Department of Biochemistry, Molecular Biology and Biophysics, University of Minnesota, Minneapolis, MN 55455, USA
[3] Department of Medicinal Chemistry, University of Minnesota, Minneapolis, MN 55455, USA
* Correspondence: ddt@umn.edu

Supplementary Materials

Supplementary Table S1: Physico-chemical cluster analysis

Compound ID	Chemical and molecular name	Chemical structure	Molecular weight (g/mol)	cLogP	Rotatable bonds	tPSA	H donors	H acceptors
		**Cluster 1**						
**DS67200668 (1)**	*N*-(4-methylbenzyl)-*N*-(1,3-thiazol-2-ylmethyl)butan-2-amine $C_{16}H_{22}N_2S$	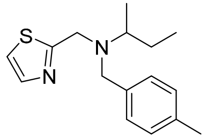	274.4	3.9	6	16	0	2
**DS75082844 (2)**	*N*-[(2-methoxypyrimidin-5-yl)methyl]-2-methyl-*N*-[(3-methyl-2-thienyl)methyl]propan-1-amine $C_{16}H_{23}N_3OS$	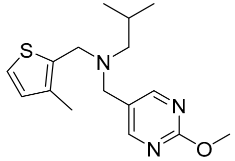	305.4	3.8	7	38	0	4
**DS76145530 (3)**	*N*-cyclopentyl-*N*-[(5-methyl-2-thienyl)methyl]-2-(propylthio)acetamide $C_{16}H_{25}NOS_2$	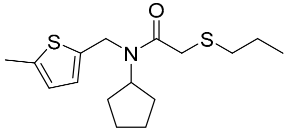	311.5	4.5	7	20	0	1
**DS81260867 (4)**	*N*-(2-methoxybenzyl)-4,5-dimethyl-*N*-propylthiophene-2-carboxamide $C_{18}H_{23}NO_2S$	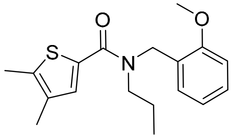	317.4	4.5	6	30	0	2
**DS11966966 (5)**	*N*-allyl-*N*-(2-ethoxybenzyl)cyclobutanecarboxamide $C_{17}H_{23}NO_2$	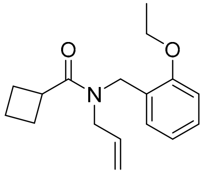	273.4	3.1	7	30	0	2
**DS25365366 (6)**	*N*-butyl-3-methyl-*N*-(3-thienylmethyl)-2-furamide $C_{15}H_{19}NO_2S$	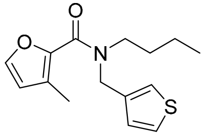	277.4	3.7	6	33	0	2

Compound ID	Chemical and molecular name	Chemical structure	Molecular weight (g/mol)	cLogP	Rotatable bonds	tPSA	H donors	H acceptors
		**Cluster 2**						
**DS31221514 (7)**	Ethyl 1-(1-cyclohexen-1-ylacetyl)-4-(3-phenylpropyl)-4-piperidinecarboxylate $C_{25}H_{35}NO_3$	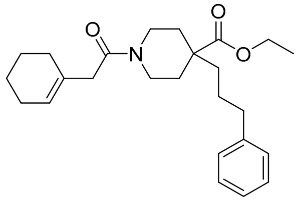	397.6	4.7	7	47	0	3
**DS39746187 (8)**	Ethyl 1-[(3-methyl-2-thienyl)carbonyl]-4-[3-(trifluoromethyl)benzyl]-4-piperidinecarboxylate $C_{22}H_{24}F_3NO_3S$	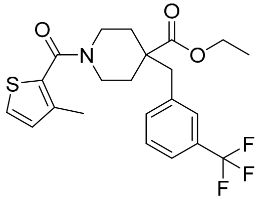	439.5	4.4	4	47	0	3
**DS84875791 (9)**	Ethyl 1-(3-methyl-2-furoyl)-4-(2-phenylethyl)-4-piperidinecarboxylate $C_{22}H_{27}NO_4$	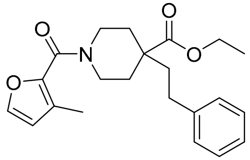	369.5	3.3	5	60	0	4
		**Cluster 3**						
**DS19784159 (10)**	3-(1,3-benzodioxol-5-yl)-*N*-methyl-*N*-[(5-methyl-2-furyl)methyl]-3-phenylpropanamide $C_{23}H_{23}NO_4$	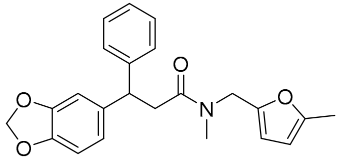	377.4	3.7	6	52	0	4
**DS27035959 (11)**	3-(1,3-benzodioxol-5-yl)-*N*-methyl-*N*-[(3-methyl-2-thienyl)methyl]-3-phenylpropanamide $C_{23}H_{23}NO_3S$	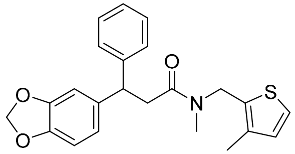	393.5	4.2	6	39	0	3

Compound ID	Chemical and molecular name	Chemical structure	Molecular weight (g/mol)	cLogP	Rotatable bonds	tPSA	H donors	H acceptors
**DS60405307 (12)**	1-{[3-(diphenylmethyl)-1,2,4-oxadiazol-5-yl]methyl}-4-(4-fluorophenyl)-1,4-diazepane $C_{27}H_{27}FN_4O$	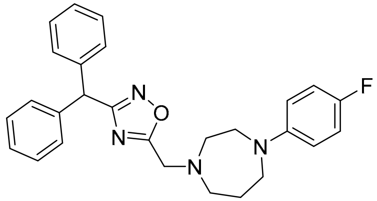	442.5	4.8	6	45	0	4
			**Cluster 4**					
**DS71721828 (13)**	*N*-({2-[benzyl(methyl)amino]pyridin-3-yl}methyl)-3-chloropyridin-2-amine $C_{19}H_{19}ClN_4$	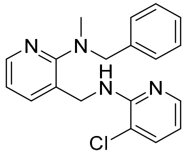	338.8	4.4	6	41	1	2
**DS32014423 (14)**	6-tert-butyl-*N*-(2-methoxybenzyl)-1-methyl-1*H*-pyrazolo[3,4-d]pyrimidin-4-amine $C_{18}H_{23}N_5O$	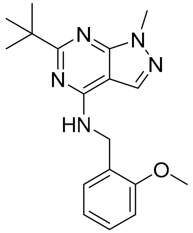	325.4	3.0	5	65	1	4
			**Cluster 5**					
**DS19396790 (15)**	3-[(2-methylprop-2-en-1-yl)oxy]-*N*-[2-(1*H*-pyrazol-1-yl)ethyl]benzamide $C_{16}H_{19}N_3O_2$	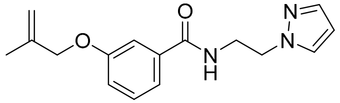	285.3	2.6	7	56	1	3
**DS33804556 (16)**	7-chloro-*N*-[2-(4-methoxyphenyl)ethyl]-3,5-dimethyl-1-benzofuran-2-carboxamide $C_{20}H_{20}ClNO_3$	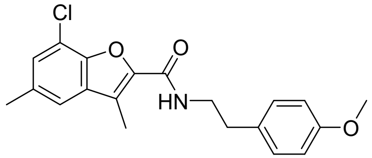	357.8	5.0	4	51	1	3
**DS39779602 (17)**	*N*-(3-ethylphenyl)-*N'*-[(4-methyl-1,2,3-thiadiazol-5-yl)methyl]succinamide $C_{16}H_{20}N_4O_2S$	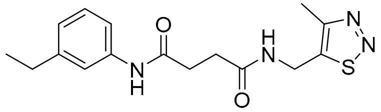	332.4	1.6	7	84	2	4

Compound ID	Chemical and molecular name	Chemical structure	Molecular weight (g/mol)	cLogP	Rotatable bonds	tPSA	H donors	H acceptors
				**Cluster 6**				
**DS81801810 (18)**	*N*-{[3-(3-methoxyphenyl)-1-phenyl-1H-pyrazol-4-yl]methyl}-*N*-[(1,3,5-trimethyl-1H-pyrazol-4-yl)methyl]cyclopropanamine $C_{27}H_{31}N_5O$	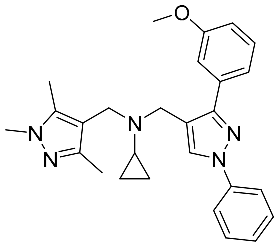	441.6	4.5	7	48	0	6
				**Cluster 7**				
**DS30616130 (19)**	(2-{5-[3-(2-phenylethyl)-1,2,4-oxadiazol-5-yl]pyridin-2-yl}phenyl)methanol $C_{22}H_{19}N_3O_2$	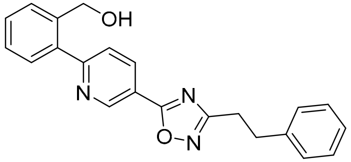	357.4	3.7	6	72	1	5

Supplementary Figure S1: Complete CRC Curves

**Compound Response Curves of Ca Uptake effect (black) and ATPase effect (red) for all compounds. V_{max} and EC50 of these curves is reflected in Table 1 of the main body of the manuscript.*

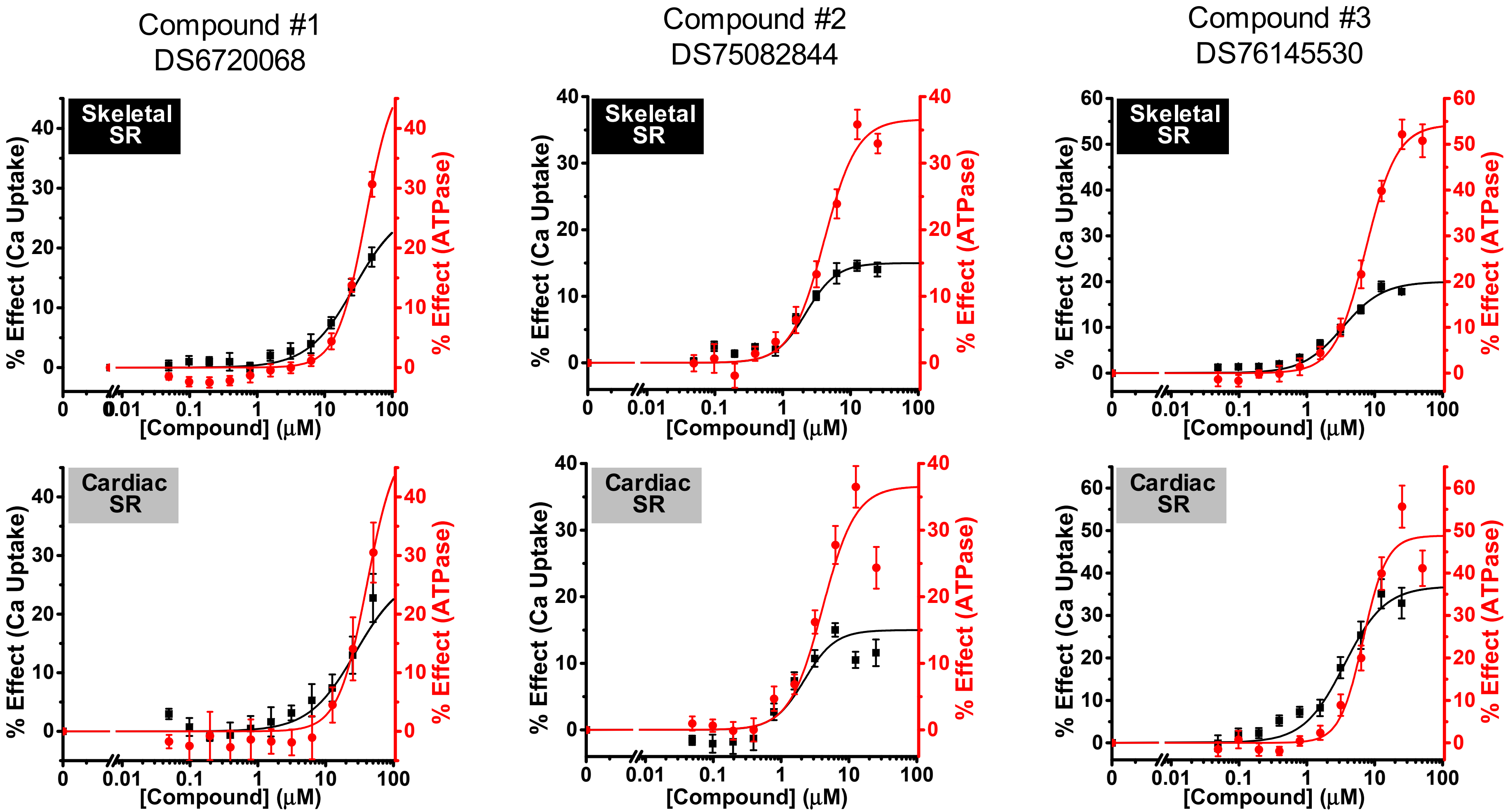

Supplementary Figure S1 Complete CRC Curves

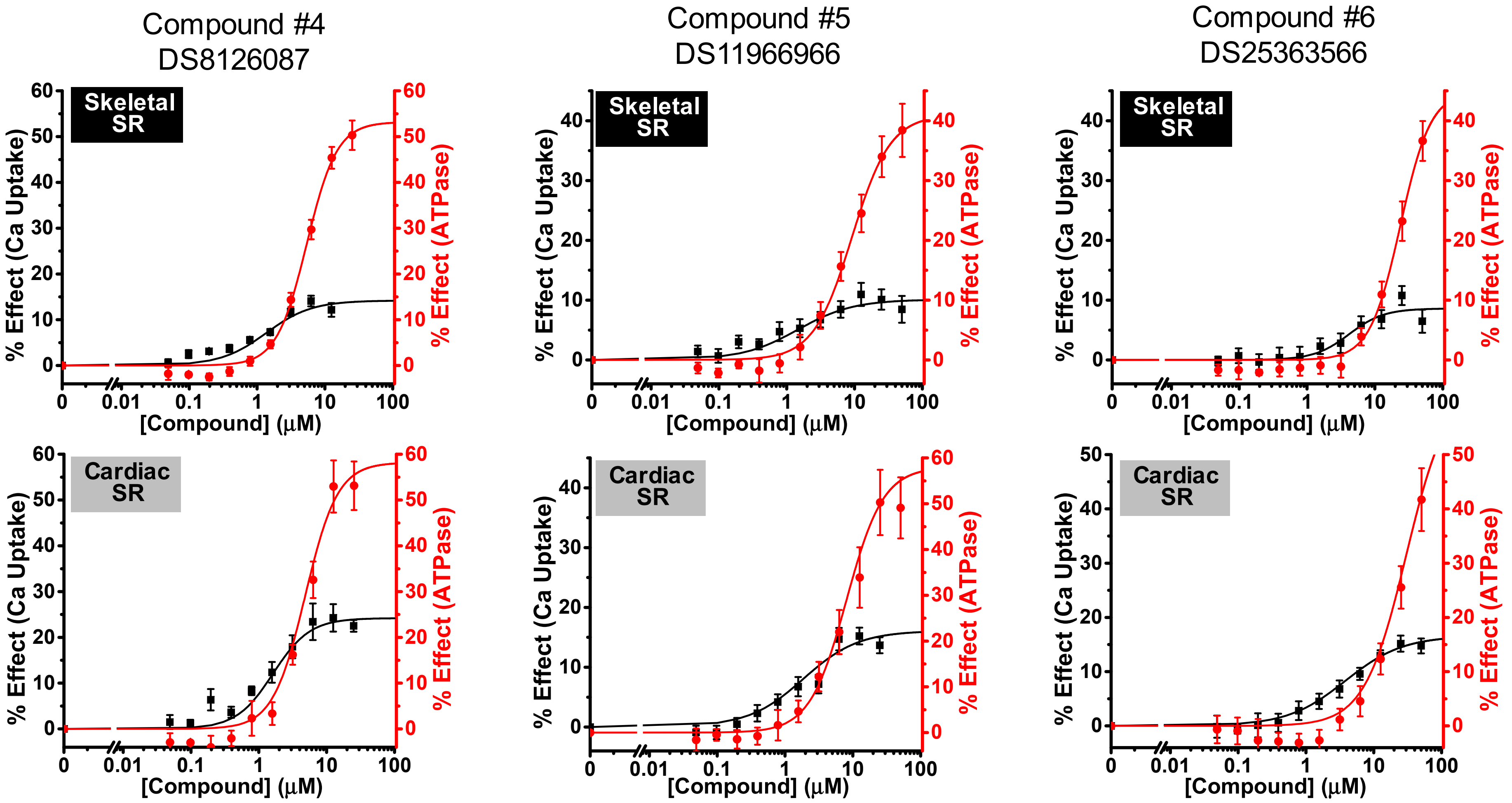

.

Supplementary Figure S1: Complete CRC Curves

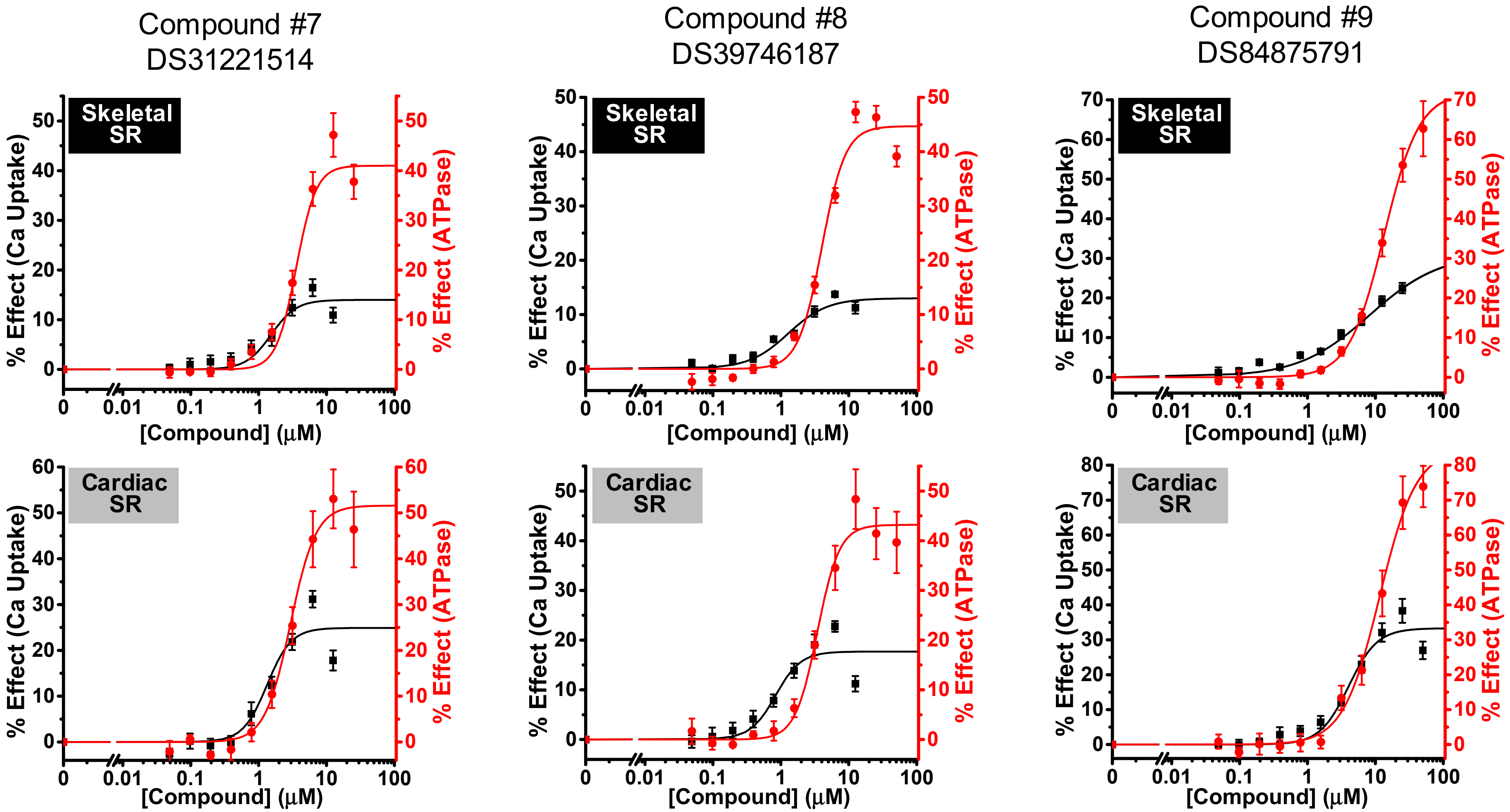

Supplementary Figure S1: Complete CRC Curves

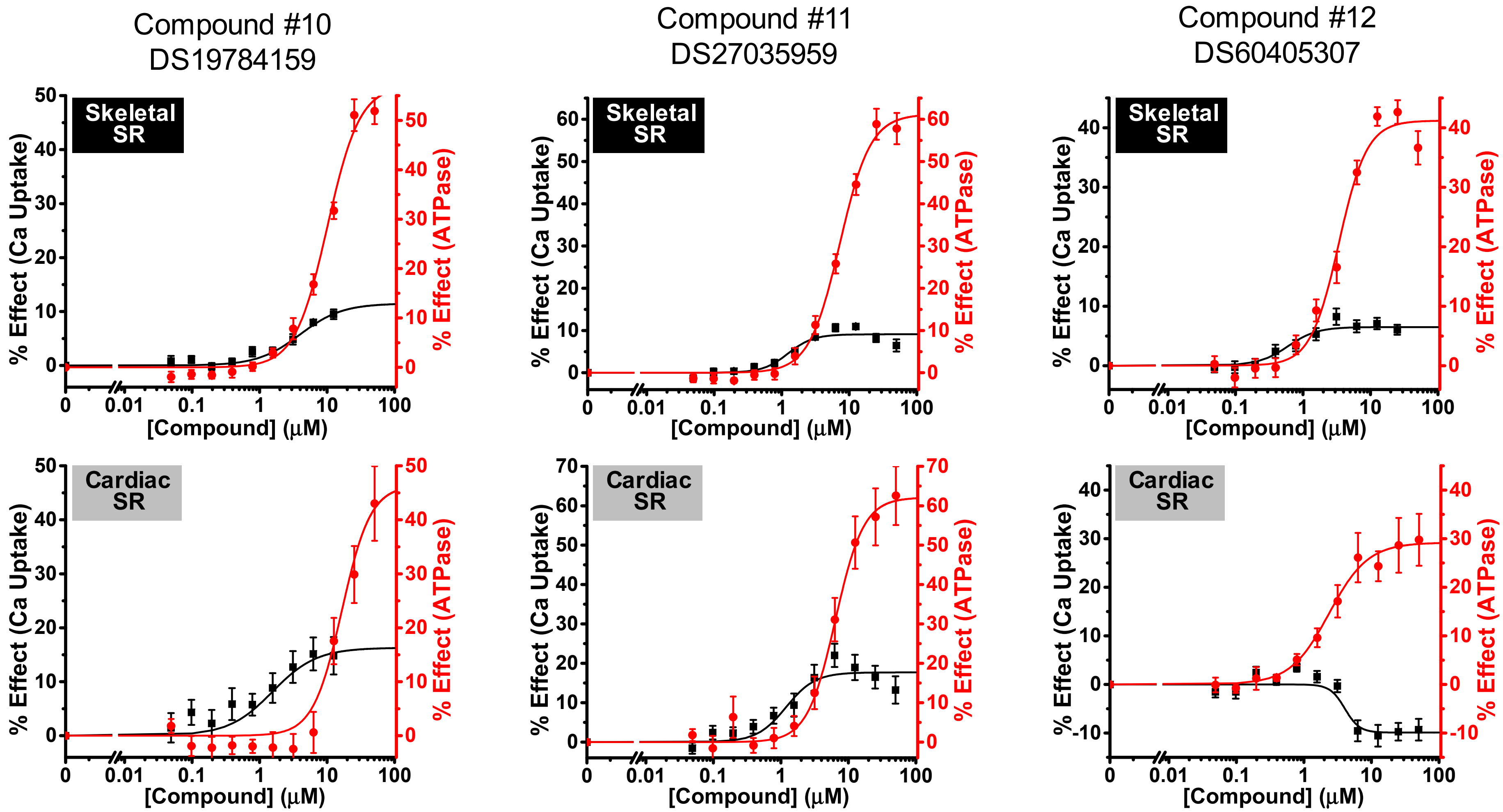

Supplementary Figure S1: Complete CRC Curves

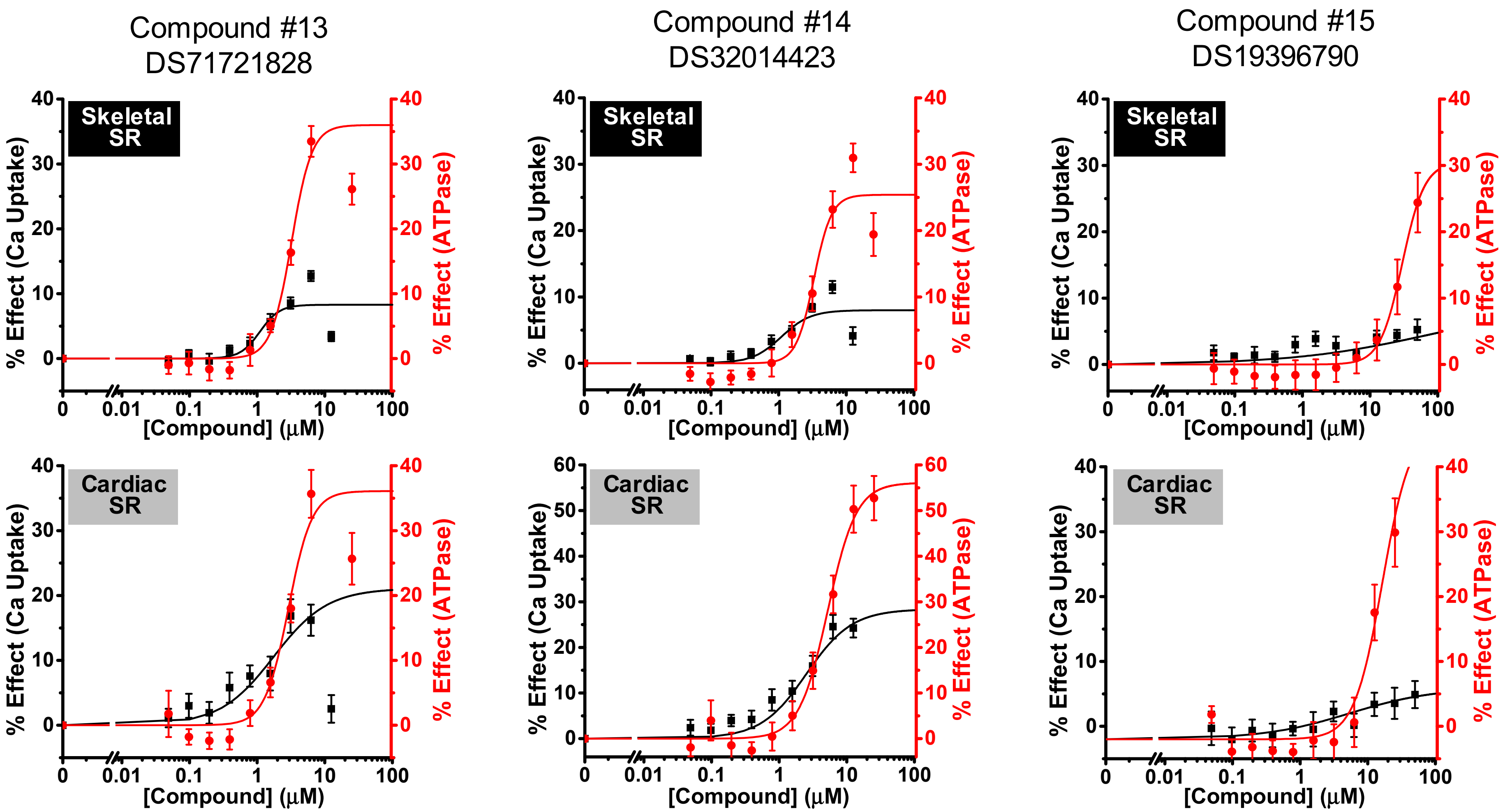

Supplementary Figure S1: Complete CRC Curves

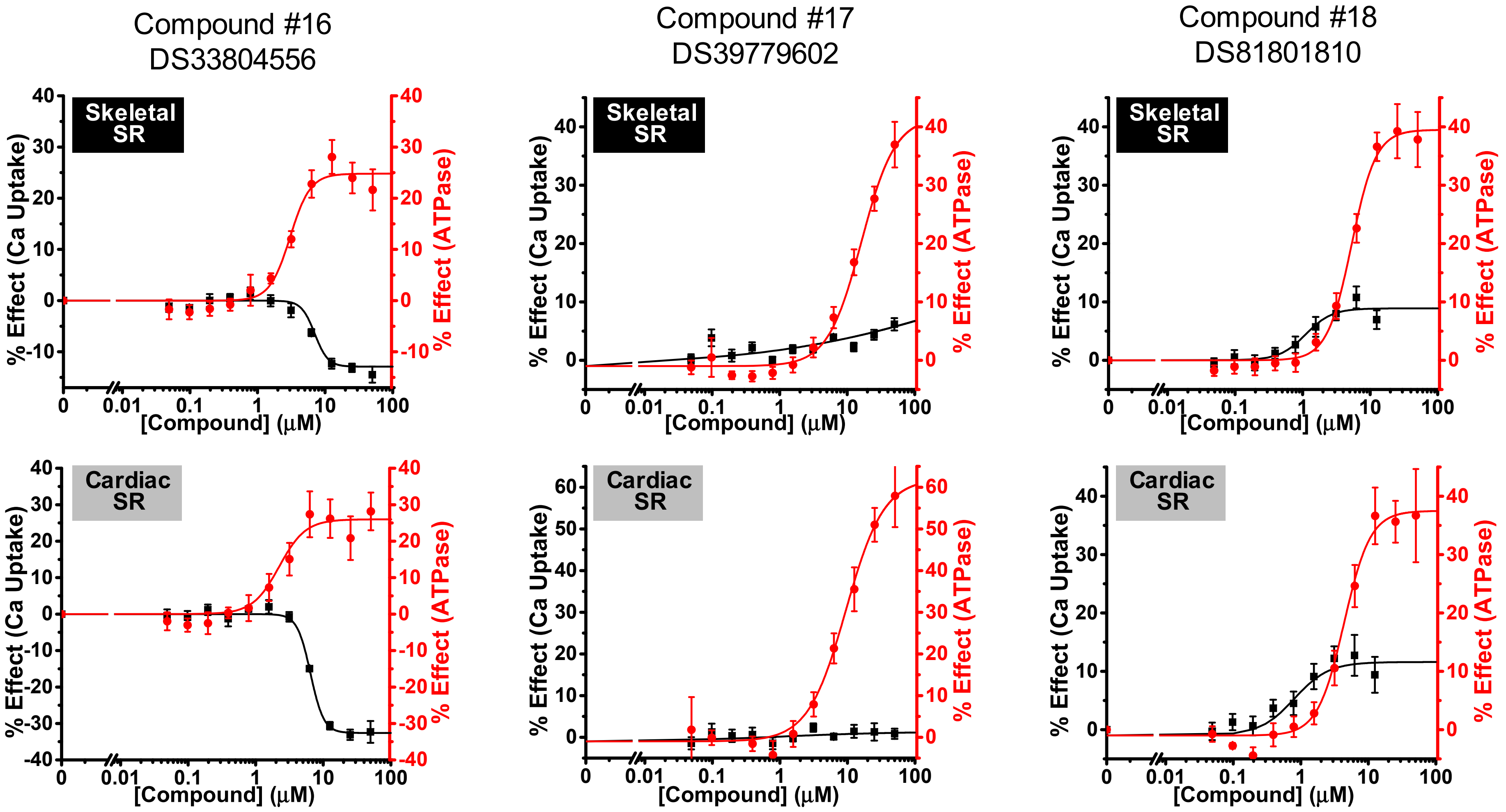

Supplementary Figure S1: Complete CRC Curves

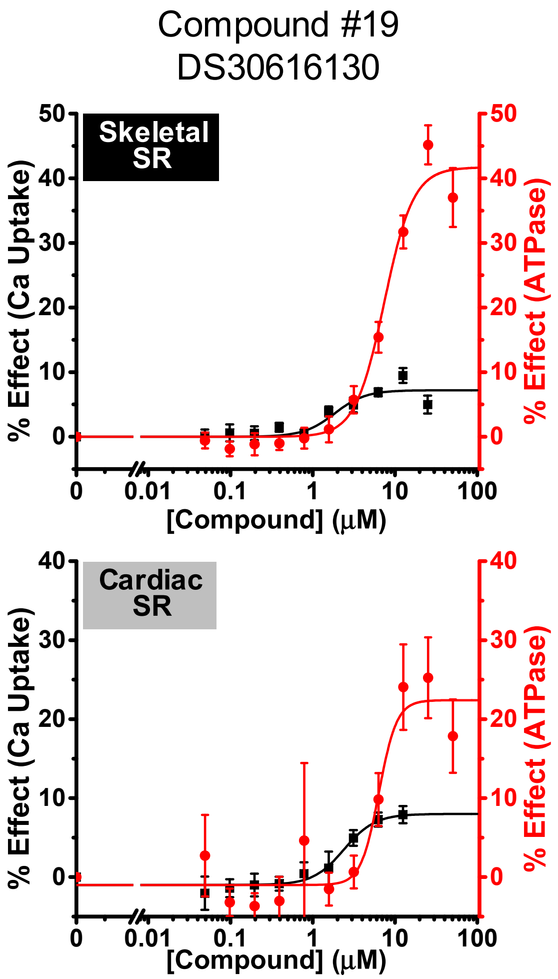

Supplement: Supplementary file 1 [file biomolecules-12-01789-s001.zip › biomolecules-2036051-supplementary.pdf]
